# Deep Learning and Cardiovascular Diseases: An Updated Narrative Review

**DOI:** 10.3390/jcm15083053

**Published:** 2026-04-16

**Authors:** Angelika Myśliwiec, Dorota Bartusik-Aebisher, Marvin Xavierselvan, Avijit Paul, David Aebisher

**Affiliations:** 1 Department of Biochemistry and General Chemistry, Medical Faculty, Collegium Medicum, University of Rzeszów, 35-310 Rzeszów, Poland; amysliwiec@ur.edu.pl (A.M.); dbartusikaebisher@ur.edu.pl (D.B.-A.); 2 Department of Biomedical Engineering, Tufts University, Medford, MA 02155, USA; marvin.xavierselvan@tufts.edu (M.X.); avijit.paul@tufts.edu (A.P.); 3 Department of Photomedicine and Physical Chemistry, Medical Faculty, Collegium Medicum, University of Rzeszów, 35-310 Rzeszów, Poland

**Keywords:** deep learning, cardiovascular diseases, AI, computational imaging techniques

## Abstract

**Background**: Artificial intelligence (AI) and deep learning (DL) are rapidly changing the field of diagnostics and imaging in cardiology, offering tools for automatic segmentation, quantification of changes, and risk stratification. These technologies have the potential to increase diagnostic accuracy, work efficiency, and individualization of patient care. **Methods**: This structured narrative review critically evaluates clinically validated applications of artificial intelligence (AI) and deep learning (DL) in cardiovascular medicine, focusing on imaging (echocardiography, coronary CT angiography, cardiac MRI, and ECG), risk stratification, and biomarker integration. A systematic literature search was conducted in PubMed for studies published between January 2015 and December 2026, supplemented by references from key articles. Original English-language studies reporting quantitative clinical outcomes were included, with 78 studies ultimately analyzed. **Results**: AI and DL models, including convolutional neural networks and transformers, achieved performance comparable to experts in cardiac imaging, myocardial perfusion assessment, valve defect detection, and coronary event prediction. Multimodal approaches improved diagnostic accuracy and reproducibility, while explainable AI enhanced transparency and clinical confidence. Deep learning also enabled faster image acquisition and processing without compromising precision. **Conclusions**: AI and DL have transformative potential in cardiology, offering fast, accurate, and scalable diagnostic tools. The integration of multimodal data, the validation of algorithms in prospective studies, and ensuring the transparency of models are key. Future research should focus on prospective, multicenter validations and the ethical and safe implementation of AI in everyday clinical practice.

## 1. Introduction

Cardiovascular diseases (CVD) remain the leading cause of morbidity and mortality worldwide, including heart diseases not directly related to coronary artery disease and strokes. This group includes, among others, heart failure not related to coronary artery disease, cardiomyopathies, arrhythmia, heart defects, and peripheral and cerebrovascular diseases, which together account for a significant proportion of cardiovascular cases and have a major impact on morbidity and mortality. In 2019, CVD accounted for approximately 18.6 million deaths [1]. Artificial intelligence (AI) encompasses methods that enable machines to perform tasks requiring human intelligence, such as data analysis, pattern recognition, and decision-making. Among AI techniques, machine learning (ML) allows computers to learn from data and independently create predictive models, while deep learning (DL), including convolutional neural networks (CNNs) and transformer-based networks, enables the processing of large and complex datasets, such as medical images or biological signals, to identify subtle patterns and biomarkers [2,3,4,5,6,7]. Major risk factors include smoking, hypertension, diabetes, obesity, and dyslipidemia [8]. At the molecular level, oxidative stress, inflammatory signaling, and mitochondrial dysfunction play a significant role in the progression of cardiovascular diseases [9].

**Figure 1:**
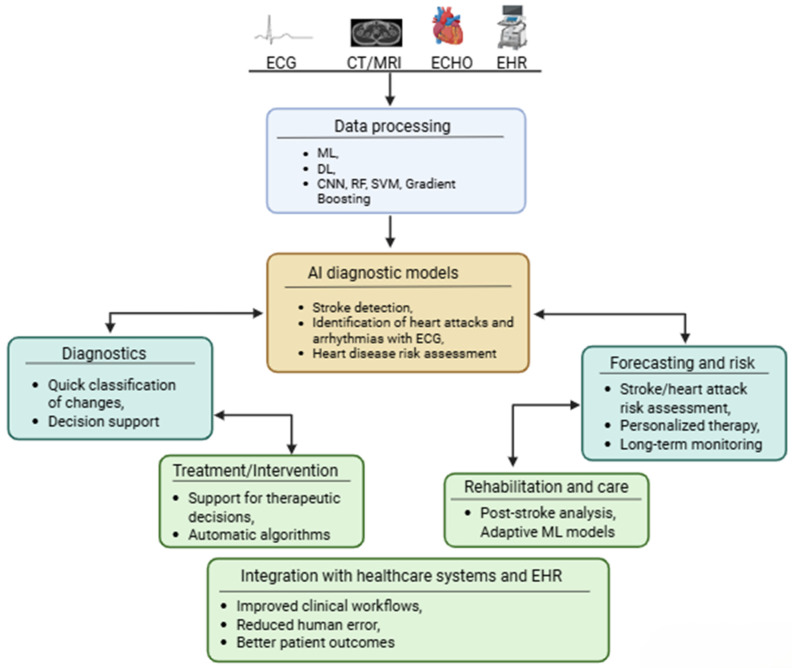
Overview of the AI (ML/DL) pipeline in cardiovascular and cerebrovascular medicine. Multimodal clinical data (ECG, CT/MRI, echocardiography, electronic health records) are processed through machine learning and deep learning algorithms (CNN, random forest, SVM, gradient boosting) to support diagnostic models for stroke detection, arrhythmia identification, and cardiovascular risk assessment. Downstream clinical applications include diagnostics, treatment support, risk forecasting, rehabilitation, and integration with healthcare systems.

Traditional risk assessment methods, such as the Framingham Risk Score, have limited accuracy and ability to personalize prognosis (Figure 1). In recent years, the development of artificial intelligence (AI) and machine learning (ML) has created new opportunities for analyzing complex clinical, demographic, and biochemical data, including lipid parameters, to predict the risk of cardiovascular events and treatment efficacy [2,3]. AI-based models, including Random Forest and Gradient Boosting algorithms, demonstrate better agreement with directly measured LDL-C concentrations than traditional formulas, making them a more reliable tool for risk assessment [2,3].

In the context of predicting heart attacks and strokes, AI enables the integration of clinical, imaging, and biochemical data, as well as the identification of new biomarkers and predictive features that are not visible to the human eye [4,5,6,7]. Deep learning (DL), particularly convolutional neural networks (CNNs), enables the automated analysis of CT, MRI, and echocardiography images, supporting the personalization of diagnosis and therapy [5,7].

The aim of this review is to critically evaluate clinically validated applications of artificial intelligence and deep learning in cardiovascular medicine, with a specific focus on their roles in imaging (echocardiography, coronary CT angiography, cardiac magnetic resonance, and electrocardiography), risk stratification, and integrated biomarker analysis, and to assess their impact on diagnostic accuracy, prognostic value, and personalized patient management.

## 2. Materials and Methods

This manuscript was designed as a structured narrative review with elements of a systematic literature search, rather than a formal systematic review, as its primary objective was to provide a broad overview of current applications of AI and DL in various areas of cardiology, including diagnostics, imaging, procedure planning, and clinical decision support.

The literature review was conducted primarily using the PubMed database to identify publications on the application of AI and DL in cardiovascular diagnostics, imaging, and clinical decision support. Additionally, the references cited in selected review articles and primary studies were analyzed to supplement the identification of relevant publications and reduce the risk of omitting important data. The search included publications released between January 2015 and December 2026, which corresponds to the period of the most dynamic development of deep learning methods in cardiovascular medicine.

The search strategy was based on a combination of MeSH terms and keywords using Boolean operators. An example search strategy used the following syntax: (“artificial intelligence” OR “deep learning” OR “machine learning”) AND (“cardiology” OR “cardiovascular disease”) AND (“echocardiography” OR “cardiac MRI” OR “cardiac CT” OR ‘CCTA’ OR “PET/CT” OR “image analysis” OR “surgical planning” OR “intraoperative navigation”).

Only original scientific papers published in English were eligible for analysis, including prospective, retrospective, cohort, observational, and interventional clinical trials. Review articles, commentaries, editorials, conference abstracts without full primary data, purely simulation studies, theoretical works without clinical validation, and studies unrelated to cardiological applications were excluded from the analysis.

Studies meeting the following criteria were included in the review: the use of AI or DL methods in diagnosis, imaging, or clinical decision-making in cardiology; an evaluation conducted in a patient population; reporting of quantitative results, such as sensitivity, specificity, area under the curve (AUC), segmentation accuracy, image reconstruction efficiency, or clinical impact.

The publication selection process consisted of two stages. In the first stage, two independent reviewers analyzed the titles and abstracts of the articles. Subsequently, the full texts of potentially eligible publications were subjected to a detailed evaluation. Disagreements were resolved through discussion, and in cases of dispute, a third reviewer was consulted for a decision.

The initial search identified 412 records. After removing duplicates and rejecting publications that did not meet the criteria during the title and abstract assessment stage, 126 articles were selected for full-text analysis, of which 78 studies were ultimately included in the qualitative synthesis of results. Across the 78 studies included in the qualitative synthesis, the cumulative study population exceeded 120,000 participants, although individual sample sizes varied substantially depending on study design, imaging modality, and clinical endpoint.

Due to the significant heterogeneity of the research designs, imaging methods used, AI architectures, and reported endpoints, a formal meta-analysis was not conducted. Instead, a qualitative thematic synthesis was applied.

Information regarding the study design, characteristics of the study population, type of AI/DL architecture used, imaging method employed, validation strategy, and main clinical outcomes was extracted from the selected publications.

The results were grouped by main thematic areas: modern computational imaging techniques, methods inspired by brain function, AI in the reconstruction and analysis of clinical images, multimodal data integration, applications in surgical planning and robotic navigation, deep learning in the interpretation of cardiovascular images, ethical aspects, transparency, and interpretability of AI models.

Methodological quality and potential risks of systematic error were assessed descriptively, taking into account the study design, sample size, model validation methods, the presence of external validation, and the reproducibility of the AI methods used. Particular attention was paid to the risk of model overfitting, limitations arising from single-center datasets, and the lack of independent external validation, which remain significant limitations of current research on AI in cardiology.

**Figure 2:**
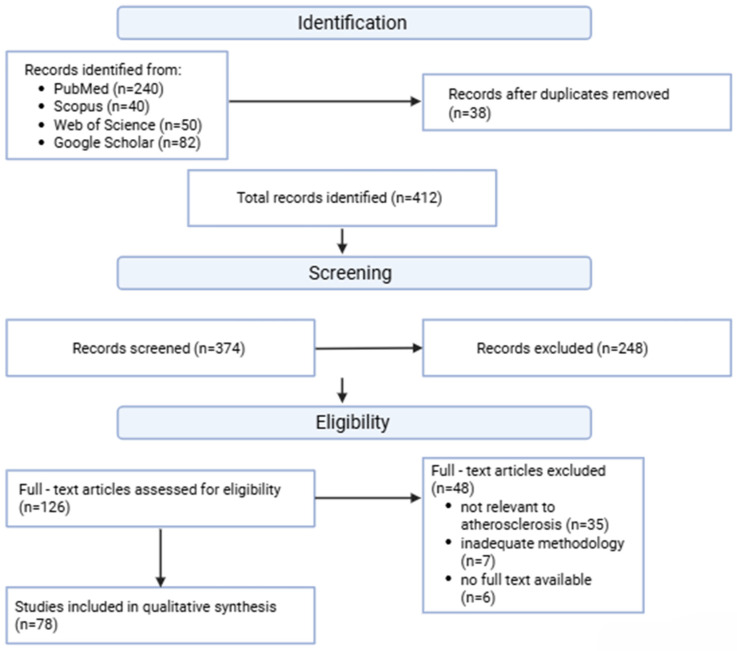
PRISMA-adapted flow diagram of literature identification and study selection. Records were identified through searches of PubMed, Scopus, Web of Science, and Google Scholar. After duplicate removal, title and abstract screening was performed independently by two reviewers, followed by full-text eligibility assessment according to predefined inclusion and exclusion criteria. Seventy-eight studies were ultimately included in the qualitative synthesis.

This approach has made it possible to provide a comprehensive overview of current research trends, methodological limitations, and potential directions for the further development of artificial intelligence applications in cardiology (Figure 2).

## 3. Results

### 3.1. Novel Computational Imaging Techniques for Enhanced Biomedical Diagnostics

Modern biomedical diagnostics is undergoing a profound transformation thanks to the integration of innovative computational imaging techniques with advanced data analysis methods. Traditional imaging methods such as CT, MRI, and PET have long been the foundation of disease detection and monitoring. Their diagnostic value is now significantly enhanced by AI and ML, which enable automatic analysis of large image datasets, improve reconstruction quality, reduce artifacts, accelerate detection of pathological changes, and increase overall diagnostic accuracy [8,9]. AI also facilitates multimodal data integration—combining clinical, imaging, and prognostic information—which allows for more personalized diagnostic and treatment pathways [8,10,11].

Recent research demonstrates the effectiveness of AI-based methods across a variety of imaging modalities, particularly in cerebrovascular and cardiovascular applications. In cerebrovascular imaging, Romagos et al. investigated the use of convolutional neural networks (CNNs) for detecting arteriovenous malformations in pediatric arterial spin labeling (ASL) MRI. Their study compared three CNN architectures for both detection and explanation generation, finding that simpler CNNs achieved the highest accuracy of 90% and produced heat maps closely matching expert assessments. This highlights the importance of balancing model complexity with clinical utility [12].

Shen et al. conducted a systematic review of ischemic stroke detection using non-contrast computed tomography (NCCT), analyzing 38 studies comprising 74 analytical samples. AI models demonstrated high sensitivity and specificity (91.2% and 96.0%) in internal validation; however, performance declined in external validation, underscoring the need for method standardization and robust external testing. The use of AI as a decision support tool improved physician performance, raising sensitivity to 83.7% and specificity to 86.7% [13]. Similarly, Soltanpour et al. applied the MultiRes U-Net model for automatic segmentation of ischemic lesions on CT perfusion maps, enriching standard images with contralateral hemisphere and Tmax maps. This approach improved Dice similarity to 0.68 compared to 0.55 for radiologists, allowing more precise delineation of stroke cores [14].

Deep learning models integrating MRI diffusion-weighted imaging (DWI) and apparent diffusion coefficient (ADC) data have further advanced ischemic lesion analysis, achieving ICC > 0.98 and Dice > 0.85, outperforming single-modality solutions and supporting highly accurate infarct core delineation [15,16,17]. Moreover, CNN models using only DWI data for the prediction of final infarct volume reached a median AUC of ~0.91, enabling faster patient triage without requiring additional perfusion imaging [18,19]. These findings collectively demonstrate the potential of AI to enhance cerebrovascular diagnostics, optimize workflow, and support clinical decision-making.

In cardiovascular imaging, coronary computed tomography angiography (CCTA) combined with fractional flow reserve (FFR-CT) has become a key tool for non-invasive functional assessment of atherosclerotic lesions, improving risk stratification and treatment planning. AI and ML models using quantitative CCTA data have been shown to outperform traditional visual assessments in predicting ischemia, achieving an AUC of 0.92, and in forecasting cardiovascular events with a C-index of 0.87 [20,21,22,23]. These approaches highlight the potential for AI-driven analysis to personalize therapy, identify high-risk patients earlier, and optimize clinical decision-making in cardiovascular care (Table 1).

### 3.2. Brain-Inspired AI Methodologies and Their Clinical Implications in Cardiovascular Diseases

The heart–brain axis reflects complex bidirectional interactions between the nervous and cardiovascular systems, in which dysfunction in one organ may influence the function and prognosis of the other. These interactions are increasingly recognized in conditions such as arrhythmia, stroke, heart failure, and autonomic dysregulation, highlighting the need for integrated diagnostic and therapeutic approaches [24]. Artificial intelligence (AI) offers powerful analytical tools for exploring large multimodal datasets, enabling detection of latent predictive patterns and supporting earlier intervention in both cardiovascular and neurological disorders [25,26,27,28,29,30,31].

AI applied in clinical medicine includes several complementary learning paradigms. Supervised learning relies on labeled datasets to develop predictive models capable of estimating outcomes in previously unseen cases. Unsupervised learning identifies latent structures and hidden associations within unlabeled datasets, supporting subgroup discovery and pattern recognition. Reinforcement learning introduces adaptive optimization through iterative interaction with an environment, where model decisions are refined according to reward-based feedback. Together, these approaches allow AI systems to process heterogeneous clinical information and support data-driven medical decision-making.

**Figure 3:**
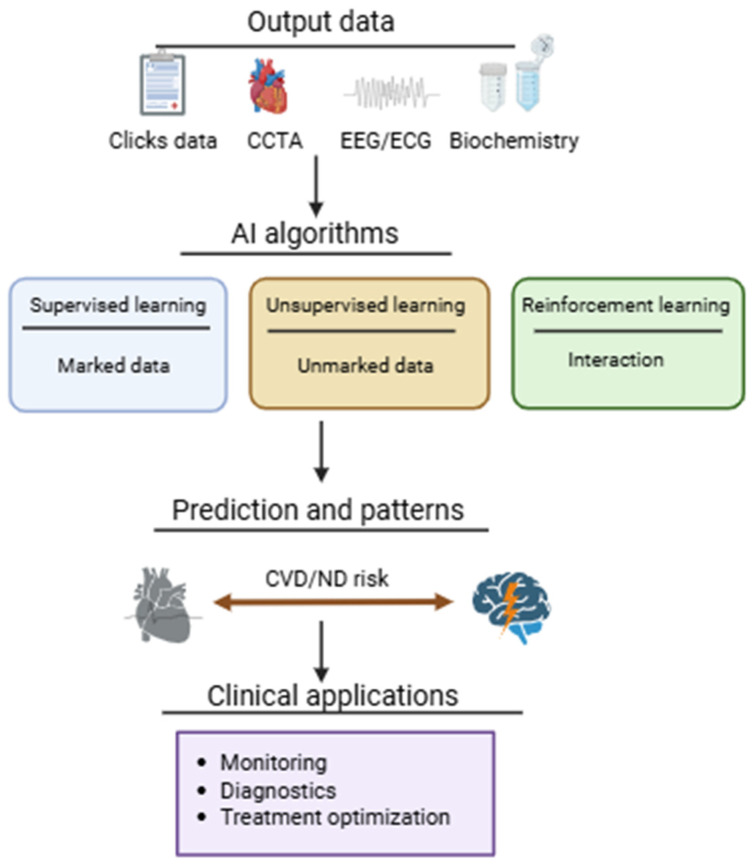
ML/DL workflow for cardiovascular and neurological disease prediction. Clinical input data, including imaging (CCTA), electrophysiology (EEG/ECG), biochemistry, and clinical records, are processed through supervised learning (labeled data), unsupervised learning (unlabeled data), or reinforcement learning (agent–environment interaction) to generate cardiovascular and neurological disease risk predictions, with downstream clinical applications in monitoring, diagnostics, and treatment optimization. CVD, cardiovascular disease; ND, neurological disease.

Brain-inspired AI architectures derive conceptual inspiration from general principles of hierarchical and distributed information processing observed in biological neural systems, without implying direct correspondence to specific anatomical regions. Architectures such as multilayer perceptrons (MLPs), convolutional neural networks (CNNs), recurrent neural networks (RNNs), and spiking neural networks (SNNs) differ in their handling of spatial, temporal, and event-based information, reflecting selected computational features of neural signal organization rather than direct mapping to defined brain structures (Figure 3). In cardiovascular medicine, these architectures support feature extraction, temporal signal interpretation, and multimodal integration across imaging, electrophysiological, and clinical datasets [32,33].

Clinical applications of these AI architectures demonstrate significant translational potential in both cardiovascular and neurocardiological settings. Artificial neural network models integrating clinical variables and circulating biomarkers have been used to predict neurological outcomes after out-of-hospital cardiac arrest (OHCA), achieving AUROC values up to 0.95 and outperforming conventional logistic regression models and established clinical risk scores [34,35,36,37,38,39,40,41,42,43]. In cardiovascular disease risk prediction, models such as DeepSurv and nonlinear time-to-event algorithms have consistently demonstrated stronger prognostic performance than Cox regression and random survival forest approaches, improving identification of high-risk patient subgroups [44].

Comparable advantages have also been observed in multimodal cardiovascular prediction. Convolutional neural network models using late-fusion strategies to combined ECG, CCTA, and clinical variables achieved higher predictive performance than classical machine learning methods, particularly in complex risk stratification scenarios where multiple physiological domains contribute simultaneously to prognosis [45].

The development of explainable artificial intelligence (XAI) further strengthens the clinical relevance of these systems by improving interpretability of model outputs, increasing clinician trust, and supporting precision medicine strategies in cardiovascular care [46]. Methods that identify feature importance and visualize decision pathways are particularly important in multimodal cardiovascular models, where transparency is essential for safe clinical implementation.

Collectively, current evidence indicates that biologically inspired AI architectures can substantially support diagnostics, prognostics, and individualized treatment planning in cardiovascular and neurocardiological diseases. However, further multicenter validation and workflow integration remain necessary before routine adoption.

### 3.3. AI-Driven Image Reconstruction, Enhancement, and Analysis in Cardiovascular Clinical Settings

Advances in cardiovascular imaging, combined with artificial intelligence (AI), are transforming the diagnosis and monitoring of heart disease. Deep learning (DL) and neural network-based tools are increasingly applied to cardiac magnetic resonance imaging (CMR), CCTA, and echocardiography for image reconstruction, quality improvement, and automated analysis. AI enables artifact reduction, denoising, faster acquisition, and multimodal data integration, which improves workflow efficiency while maintaining or enhancing diagnostic performance [47,48].

**Figure 4:**
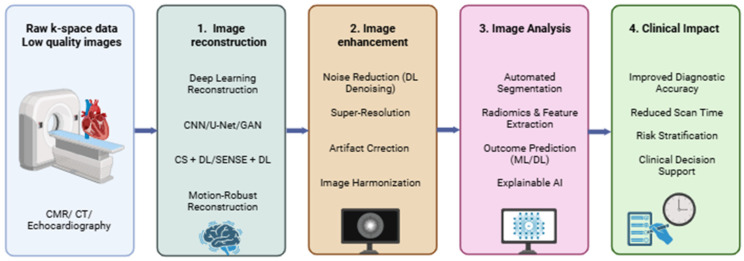
Four-stage AI-driven pipeline for cardiovascular image processing. Stage 1 (Image Reconstruction): raw k-space data from CMR, CT, and echocardiography are reconstructed using deep learning methods including CNN, U-Net, GAN, and compressed sensing combined with DL. Stage 2 (Image Enhancement): noise reduction, super-resolution, artifact correction, and image harmonization. Stage 3 (Image Analysis): automated segmentation, radiomics and feature extraction, outcome prediction via ML/DL, and explainable AI. Stage 4 (Clinical Impact): improved diagnostic accuracy, reduced scan time, risk stratification, and clinical decision support.

CMR is a key non-invasive method for assessing cardiac anatomy, function, and tissue characteristics, providing excellent soft tissue contrast (Figure 4). Clinically important sequences include cine imaging for ventricular function and valve motion, T2 STIR sequences for edema detection, late gadolinium enhancement (LGE) for fibrosis and scarring, and perfusion imaging for evaluating ischemic regions [49,50,51,52,53]. These capabilities allow precise assessment of disease severity, monitoring of therapy, and prognosis. However, prolonged acquisition times, particularly in contrast-enhanced studies (40–50 min), can increase patient discomfort, motion artifacts, and limit feasibility in high-volume centers or vulnerable populations [54].

Parallel imaging techniques such as SENSE, often combined with compressed sensing (CS), accelerate acquisition but rely on iterative algorithms and sparsity assumptions, sometimes reducing image fidelity in dynamic cardiac imaging [55,56,57]. DL-based reconstruction has emerged as a solution, demonstrating simultaneous improvements in acquisition speed and image quality [54,58]. Late fusion CNN models allow the integration of multiple data sources, including ECG, tomographic images, and clinical data, improving cardiovascular event prediction by mimicking the brain’s multimodal information processing [45,59]. Explainable AI (XAI) further increases clinical trust by identifying factors driving prognosis and supporting therapeutic decisions [60,61].

AI applications in cardiac imaging now extend beyond reconstruction to automatic segmentation, myocardial infarction detection, and risk prediction. These tools enhance diagnostic accuracy, shorten examination times, and facilitate personalized care [62,63]. For instance, AI can automate slice positioning in CMR and optimize B0 field homogeneity, reducing exam time and improving signal-to-noise ratio (SNR) [64]. Similarly, U-Net and 3D-DenseNet architectures enable automatic detection of the cardiac resting period for angiographic acquisition, shortening trigger delay times and enhancing workflow [65,66].

AI has also shown promise in optimizing inversion time (TI) selection for LGE imaging. Models using CNNs and LSTM networks, or semi-automated threshold-based approaches, achieved high agreement with expert decisions, streamlining the imaging process while maintaining reproducibility [67,68,69,70]. Fully automated TI selection using U-Net further reduces reliance on manual shim positioning and, for phase-sensitive inversion recovery (PSIR) sequences, eliminates the need for TI-scouts [71,72,73,74]. Artifact reduction methods using U-Net and 3D CNNs accelerate reconstruction fivefold while preserving image quality, particularly in undersampled radial MRI sequences [75]. Hybrid methods combining DL with traditional iterative reconstruction maintain data consistency and improve final image quality [76].

Recent studies, including clinical examinations using CSAI algorithms, demonstrate that DL-based reconstruction can reduce total acquisition times by more than 50%, while improving SNR, contrast-to-noise ratio (CNR), and edge sharpness, without compromising functional measurements or diagnostic accuracy [48]. These findings indicate that AI integration into CMR and other cardiovascular imaging modalities offers substantial gains in efficiency, image quality, and clinical decision support.

Table 2 summarizes representative AI techniques applied across stages of cardiovascular imaging. CNNs, U-Net, GANs, and iterative DL algorithms are used for reconstruction, dose reduction, contrast enhancement, and automated segmentation, with clinical benefits including faster acquisition, improved signal quality, and enhanced risk prediction [48,61,77,78,79,80].

### 3.4. Multi-Modal Data Fusion Strategies for Improved Clinical Decision-Making in Cardiovascular Diseases

Medical image reconstruction and analysis play an increasingly important role in cardiovascular diagnostics, particularly in CMR. Traditional reconstruction methods such as sensitivity encoding (SENSE) and compressed sensing reduce scan time but remain limited by reconstruction complexity and potential degradation of image fidelity in demanding clinical settings. Recent advances in artificial intelligence, especially deep learning (DL), have introduced new reconstruction frameworks that shorten acquisition while preserving clinically relevant image characteristics, thereby improving workflow efficiency in cardiovascular imaging [81].

A representative example is the AI-based cine method with motion correction and free breathing (FB AI MOCO), evaluated in 72 participants. This technique significantly shortened acquisition time compared with standard breath-hold cine imaging while maintaining comparable ventricular functional assessment. Total scan time was more than five times shorter, with non-significant differences in apparent signal-to-noise ratio (aSNR), significantly improved estimated contrast-to-noise ratio (eCNR), and superior blood pool-to-myocardial contrast. Subjective image quality remained equivalent between methods, and ventricular functional parameters showed excellent agreement (ICC > 0.900), including in patients with reduced ejection fraction or elevated heart rate, supporting the feasibility of this method in routine clinical practice [82].

Beyond reconstruction, post-processing approaches have become equally important for improving the diagnostic usability of cardiovascular images. AI-based denoising, super-resolution, and artifact correction methods enhance image consistency after acquisition, particularly in CMR and CT workflows, where reduced motion sensitivity and improved contrast support more reliable interpretation [83,84,85]. These developments extend naturally into automated image analysis, where segmentation, feature extraction, and prediction models increasingly contribute to risk stratification and therapeutic planning. Because deep learning systems often function as opaque decision models, explainable artificial intelligence (XAI) techniques such as Grad-CAM and SHAP are increasingly incorporated to visualize feature relevance and improve interpretability, although methodological standardization remains limited [62,86,87].

In coronary microvascular dysfunction (CMVD), invasive coronary flow reserve measurement using Doppler wire remains the clinical reference standard, but routine use is constrained by procedural complexity and limited accessibility [88]. Non-invasive alternatives such as echocardiography [89,90], PET/CT for myocardial metabolism [91], and CMR [92,93] provide quantitative perfusion assessment, yet most current approaches evaluate isolated physiological domains rather than integrating perfusion, mechanics, and metabolism simultaneously [94,95,96]. This limitation has driven interest in multimodal analytical frameworks capable of combining complementary physiological signals into unified clinical models.

Deep learning has already demonstrated strong performance in cardiovascular image interpretation, particularly in segmentation, lesion detection, disease classification, and quantitative analysis [97]. In myocardial imaging, these methods improve reproducibility and reduce operator dependence in pathology characterization [98,99,100,101,102,103,104]. However, despite strong technical performance, clinical adoption remains restricted by concerns regarding interpretability and model transparency [105]. To address this, newer XAI-oriented frameworks combine predictive output with biologically meaningful explanations, improving potential for clinical translation.

Recent multimodal applications illustrate this trend across imaging modalities. In echocardiography, lightweight convolutional architectures and transformer-based models such as EchoNet-Dynamic and UltraViT enable real-time chamber segmentation and estimation of functional parameters including ventricular volume and ejection fraction with low computational demand [105]. In PET/CT, attention-enhanced U-Net architectures improve myocardial perfusion quantification, pathological localization, and noise control, supporting lower-dose protocols [106]. Comparable progress is observed in CMR, where nnU-Net and transformer-enhanced U-Net models achieve segmentation accuracy comparable to expert readers, facilitating repeatable myocardial assessment [107]. In nuclear magnetic resonance spectroscopy, DL approaches improve metabolite quantification and biomarker extraction, increasing sensitivity for detecting cardiovascular metabolic abnormalities [108].

These developments provide the methodological basis for multimodal diagnostic systems such as the CMVD_MDAS model, which integrates echocardiography, PET/CT, and CMR into a unified analytical framework for coronary microvascular dysfunction assessment [109]. Such systems reflect a broader transition toward data fusion strategies that combine imaging and non-imaging sources to support clinical decision-making.

In cardiovascular AI, three principal multimodal fusion strategies are currently distinguished: early fusion, late fusion, and hybrid/intermediate fusion. Early fusion combines heterogeneous data directly at the model input stage, enabling joint representation learning across modalities. This strategy has been applied successfully in post-PCI prediction models using combined ECG and electronic medical record (EMR) data, where multimodal input improved predictive performance relative to unimodal approaches [61].

**Figure 5:**
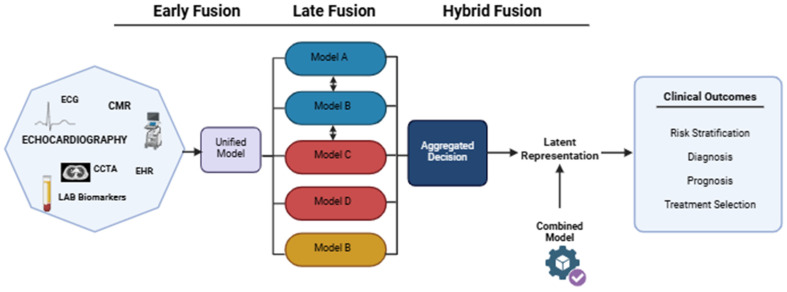
A conceptual representation of a multimodal data fusion strategy in cardiovascular artificial intelligence. Multiple clinical data sources, including ECG, echocardiography, CCTA, CMR, PET/CT, laboratory biomarkers, and electronic health records (EHRs), can be integrated using three main approaches: early fusion, in which raw features are fused as input to the model; late fusion, in which modality-specific predictions are aggregated at the decision stage; and hybrid fusion, in which intermediate, latent representations are merged before the final prediction. These strategies support clinical applications such as diagnosis, risk stratification, prognosis, and treatment planning.

To facilitate interpretation of multimodal fusion strategies, Figure 5 illustrates the principal architectures currently used for integrating heterogeneous cardiovascular data into AI-supported clinical decision models.

Late fusion processes each modality independently before combining outputs at the decision stage. This architecture offers greater robustness when some modalities are unavailable and has shown strong performance in clinical imaging tasks such as pulmonary embolism classification, where image-derived and clinical predictors were aggregated with high AUROC [110]. Hybrid fusion combines intermediate representations extracted separately from each modality, allowing preservation of complementary feature hierarchies while capturing deeper cross-modal relationships. Although hybrid systems remain less mature in cardiology, emerging studies suggest advantages in combining imaging and clinical data for coronary artery disease diagnosis [111].

The choice of fusion strategy depends on data structure, availability of modalities, robustness requirements, interpretability, and computational constraints. Early fusion facilitates low-level interaction learning but requires harmonized input data (Table 3). Late fusion offers modularity and practical resilience to missing information, whereas hybrid fusion provides richer feature interaction at the cost of greater architectural complexity. Strategic use of these architectures enables clinically useful diagnostic systems that balance predictive strength, reproducibility, and interpretability in cardiovascular disease management [59,110,111].

### 3.5. Applications of AI in Surgical Planning, Robotic Guidance, and Intraoperative Navigation in Cardiovascular Diseases

Artificial intelligence, combined with advanced medical image analysis and machine learning methods, is increasingly integrated into cardiovascular surgery planning, where it supports anatomical modeling, procedural simulation, and perioperative risk estimation. In preoperative settings, AI facilitates segmentation of anatomical structures, automated three-dimensional reconstruction, and optimization of surgical strategies based on multimodal imaging and clinical information. According to Mank et al., these approaches improve surgical preparation by reducing manual processing demands and enabling more structured planning workflows [113]. Similarly, Leivaditis et al. showed that combining imaging-derived and clinical parameters within AI-supported planning frameworks improves preoperative risk stratification and contributes to more individualized surgical decision-making [114]. Comparable principles are already being applied in coronary revascularization, where AI supports interpretation of preprocedural datasets and assists in selecting interventional strategies [115].

In parallel with preoperative planning, AI is increasingly relevant in robotic cardiovascular surgery. Deep learning-assisted robotic systems support fine motor tasks such as suture placement and instrument control in anatomically restricted thoracic regions, contributing to minimally invasive procedural execution [116]. Experimental studies further show that AI models can recognize discrete surgical phases and evaluate operator performance during simulated cardiac procedures, suggesting future applications in semi-automated robotic assistance and procedural standardization [117,118].

A further area of development is intraoperative navigation supported by AI and real-time imaging. Integration of robotics with three-dimensional image guidance and augmented reality enables dynamic visualization of anatomical landmarks during intervention, improving spatial orientation and procedural control. These systems allow continuous adaptation of intervention trajectories to patient-specific anatomy and may support the development of more personalized minimally invasive cardiovascular procedures, although broader clinical implementation still requires standardization and multicenter validation [119,120,121].

The evolution of these technologies is closely linked to predictive machine learning models used before and after intervention. In coronary artery disease (CAD), machine learning has expanded quantitative risk estimation through digital models based on electronic medical records and multimodal clinical variables [122]. In coronary intervention settings, machine learning models also outperformed conventional logistic regression in predicting in-hospital mortality, heart failure, and rehospitalization after percutaneous coronary intervention (PCI) [123,124]. Additional work using ECG and chest radiography demonstrated predictive utility for both medium- and long-term cardiovascular event risk in CAD populations [125,126].

Although gains over conventional NCDR-CathPCI scores for bleeding and acute kidney injury prediction remain moderate [127], combining clinical variables with proteomic biomarkers substantially improves identification of acute kidney injury risk during coronary angiography [128]. In coronary CT angiography (CCTA), deep learning—particularly convolutional neural networks—has enabled automated coronary artery segmentation, plaque analysis, and stenosis classification [129,130]. When integrated with machine learning, these image-derived features support identification of high-risk plaque phenotypes and prediction of acute coronary syndromes using parameters such as lumen diameter, lesion length, FFR-CT, lipid core characteristics, and fibrous cap morphology [131].

Radiomics-based approaches further extend CCTA interpretation by extracting texture, shape, and signal intensity features associated with unstable plaque behavior and chronic myocardial ischemia [132,133,134]. Together, these methods improve lesion phenotyping and contribute to more detailed risk stratification while reducing operator dependency in vessel analysis [135,136,137].

The integration of AI with procedural imaging is also evident in structural interventions. Kumar et al. demonstrated that combining AI-assisted preprocedural cardiac CT with intracardiac echocardiography (ICE) provides an effective alternative to transesophageal echocardiography (TEE)-guided left atrial appendage occlusion (LAAO). In a retrospective cohort of 143 patients, CT/AI + ICE planning achieved similar procedural efficacy and complication rates while significantly reducing device repositioning and recapture, suggesting more accurate implant sizing and improved procedural control [138].

Artificial intelligence also supports earlier identification of structural heart disease outside procedural environments. Poterucha et al. developed the EchoNext deep learning model using over one million ECGs linked to echocardiographic labels. Despite relying only on ECG input, the model effectively predicted structural heart disease and outperformed experienced cardiologists in identifying patients requiring echocardiographic evaluation. Its performance remained stable across external validation cohorts and diverse demographic populations, and prospective clinical testing demonstrated detection of previously unrecognized disease [139].

**Figure 6:**
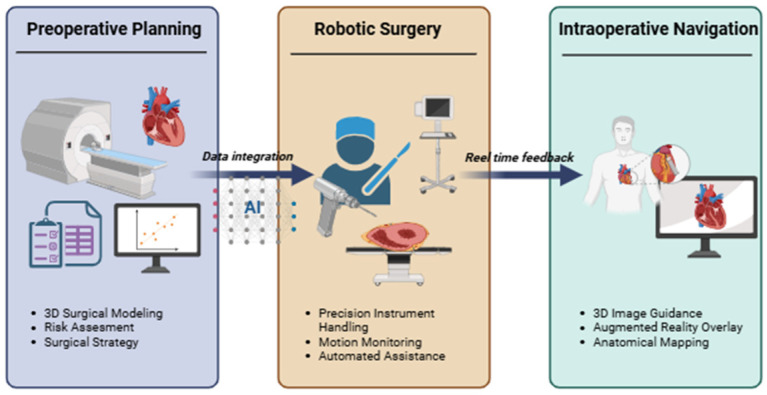
AI-supported workflow across three stages of cardiovascular surgery. Preoperative planning integrates 3D surgical modeling, risk assessment, and strategy optimization from multimodal imaging. Data are passed to robotic surgery systems for precision instrument handling, motion monitoring, and automated assistance. Real-time feedback connects to intraoperative navigation via 3D image guidance, augmented reality overlay, and anatomical mapping.

Comparable diagnostic utility has been reported in noninvasive ischemia detection. In 183 patients, the AI-QCT ISCHEMIA model, based on machine learning and convolutional analysis of CCTA, achieved sensitivity and specificity comparable to or exceeding SPECT imaging. In female patients, negative predictive value was significantly higher than SPECT (91% vs. 68%, *p* = 0.042), while overall ROC performance remained similar between methods [140]. These findings support AI-assisted CCTA as a practical noninvasive option for ischemia evaluation in low- and intermediate-risk CAD populations (Figure 6).

Predictive applications also extend to valvular heart disease. AI-ECG models trained on nearly one million paired ECG and echocardiographic examinations accurately predicted future moderate-to-severe valvular regurgitation, including mitral, tricuspid, and aortic regurgitation. These predictions correlated with early ventricular remodeling, suggesting that AI-based ECG screening may support selective referral for surveillance echocardiography and earlier intervention [141].

### 3.6. Ethical Considerations and Interpretability in AI-Driven Biomedical and Clinical Applications in Cardiovascular Diseases

With the growing use of AI in the diagnosis and treatment of cardiovascular diseases, significant ethical and interpretability issues arise that must be considered alongside clinical benefits. ML/DL models analyzing large and complex datasets can improve diagnostic accuracy and the efficiency of treatment decisions, but their inner workings often remain opaque, as highlighted by analyses of the “black box” challenges in medicine. The inability to understand how an algorithm arrives at a specific prediction can undermine clinical trust and prevent full explanation of the basis for a medical decision to the patient, which is crucial for patient autonomy and clinical accountability [142].

AI interpretability is linked to broader ethical issues, such as data privacy protection, preventing bias, and fairness in access to technology. A multidisciplinary perspective on AI model interpretability demonstrates that transparency is not merely a technical feature but a necessary condition for ethical implementation, encompassing legal, clinical, and patient-experience aspects, such as informed consent and accountability for AI-based decisions [143].

Furthermore, analysis of responsible AI in cardiovascular disease detection indicates that combining explainability tools (e.g., SHAP, LIME), privacy protection, and transparent reporting of results can support user trust and compliance with ethical principles and data protection standards [144]. Social and practical ethical aspects are also important, such as patient perceptions of AI and the potential risk of unintentionally exacerbating health inequalities. Studies from the patient perspective demonstrate that trust in AI systems depends not only on their effectiveness but also on understanding their operation, risk communication, and maintaining a “human touch” in clinical care [145]. Furthermore, the ethical literature on medical AI emphasizes the need to create regulatory frameworks and practical guidelines that ensure safety, fairness, and accountability in AI implementation in cardiology, so that the technology supports patient care without violating fundamental principles of medical ethics [146].

Beyond interpretability itself, ethical discourse surrounding AI in healthcare also encompasses algorithmic fairness and equity in the application of models across diverse patient populations. Studies have shown that AI algorithms trained on demographically unrepresentative data can lead to unequal clinical outcomes, where certain populations, including ethnic minorities or those with lower socioeconomic status, may be diagnosed or treated less accurately than others, exacerbating existing disparities in healthcare. Analysis of algorithmic fairness in medicine highlights that biases can arise at various stages of the data lifecycle—from data acquisition, through labeling, to modeling—and their effects can impact equity of access and treatment equity, posing a significant ethical challenge to the implementation of AI in clinical practice [147]. There is also an active ethical debate in the literature regarding the interpretability requirement for AI-based clinical decision support systems. A systematic analysis of the arguments indicates that although “black box” models can achieve high predictive performance, their lack of transparency raises significant concerns about accountability, informed patient consent, and the ability of physicians to monitor and respond to unexpected algorithm errors. These discussions are reflected in the ethical analysis of the explainability requirement for AI-DSS systems, where both proponents and opponents of ad-hoc or post-hoc interpretability point to different clinical contexts in which the level of explainability should be adapted to the risk and nature of the medical decision [148].

### 3.7. Limitations of Current Evidence and Translational Gaps

Despite the rapidly growing number of studies demonstrating promising applications of artificial intelligence (AI) and machine learning (ML) in cardiology, several significant methodological limitations continue to undermine the robustness, reproducibility, and practical applicability of these models in clinical practice.

One of the most serious challenges is the risk of data leakage during model development. In many studies, insufficient separation of training, validation, and test datasets can lead to overly optimistic performance estimates, particularly when images, repeat examinations, or highly correlated patient data are inadvertently shared across subsets. Such leakage can significantly overstate reported diagnostic accuracy while masking poor performance in real-world settings. For example, Verhaeghe et al. validated ML models on various independent datasets and demonstrated that only after recalibration does the model maintain performance comparable to that observed in the training data, underscoring the need for external validation in clinical trials [149].

Another significant limitation concerns the discrepancies between training datasets and real-world clinical populations. Many artificial intelligence models are developed based on retrospective, single-center datasets collected according to highly standardized imaging protocols, which often do not sufficiently reflect the diversity found in everyday clinical practice. Differences in device manufacturers, acquisition parameters, patient demographics, disease prevalence, and comorbidity profiles can significantly limit the generalizability of model results during external validation. Manini C. et al. demonstrate that the quality and diversity of training data—including representation of different populations, devices, and clinical conditions—are critical to whether a model will perform effectively beyond the training data [150].

Many studies on artificial intelligence in cardiology still do not devote sufficient attention to model calibration. High discriminatory metrics, such as AUC, do not necessarily indicate accurate probability estimates, which are essential for clinical decision-making. Poorly calibrated models can generate misleading risk estimates, potentially influencing treatment decisions, particularly in prognostic applications such as predicting cardiovascular events or assessing mortality. A review of the literature on machine learning in medical imaging has shown that only a small percentage of studies routinely perform external validation of their models, raising serious doubts about their generalizability and actual clinical utility [151]. A recurrent concern across the literature is the absence of standardized benchmarking frameworks, as different studies employ varying evaluation metrics (e.g., AUC, Dice coefficient, sensitivity/specificity) and reference standards, complicating direct cross-study comparisons. Additionally, the risk of bias in many studies was not systematically assessed, and only a minority of the cited works reported results in accordance with established reporting guidelines such as TRIPOD [152] or CLAIM [153].

An additional methodological challenge is the limited interpretability of many deep learning systems. Although convolutional neural networks and transformer-based architectures often achieve high predictive performance, the decision-making mechanisms underlying them remain difficult to explain. Explainable artificial intelligence (XAI) methods, including SHAP, Grad-CAM, and importance mapping, are increasingly being used; however, these techniques are not yet standardized and may provide incomplete or inconsistent explanations. For example, in a study by Yang et al., a deep learning model for diagnosing pulmonary edema was interpreted using Grad-CAM, but the analysis revealed pitfalls and limitations regarding the alignment of the model’s explanations with actual clinical outcomes, highlighting the ongoing challenges of achieving reliable interpretability in medical AI systems [154]. The “black-box” nature of DL models also remains a significant barrier to clinical adoption; although explainability methods such as Grad-CAM and SHAP are increasingly applied, their clinical utility and reliability have yet to be rigorously validated in prospective settings.

External validation remains one of the weakest aspects of the current state of knowledge. A significant proportion of published models are evaluated exclusively internally, often using cross-validation techniques, while independent multicenter validation remains relatively rare. As a result, many of the described algorithms have not yet demonstrated sufficient robustness for routine clinical use. For example, a comprehensive review by Wessler et al. found that approximately 58% of clinical prediction models in cardiology have never undergone external validation, and even when external validation was performed, model performance often declined compared to performance during the development phase, underscoring the need for broader testing in diverse clinical settings [155].

Further barriers to implementation include: limited interoperability with hospital IT systems, regulatory requirements, uncertainty regarding medical-legal issues, and the lack of a standardized reporting framework for the development and evaluation of AI models. Furthermore, clinicians often approach AI-based decision support systems with caution when model results cannot be fully interpreted or verified against established diagnostic pathways [156]. Moreover, real-world implementation challenges—including workflow integration, regulatory approval, computational costs, and clinician trust—are underexplored in the current body of evidence [157]. Most studies evaluated AI performance under controlled experimental conditions that do not fully replicate the complexity and variability of routine clinical practice. Prospective, multicenter clinical trials with pre-registered protocols, diverse patient cohorts, and head-to-head comparisons against standard-of-care workflows are therefore essential to confirm the clinical value and safety of AI-driven tools in cardiovascular medicine.

In summary, these limitations indicate that while AI offers significant potential in cardiovascular, imaging, and risk prediction, broader clinical adoption will require rigorous prospective validation, transparent methodological reporting, multicenter external validation studies, and better integration of explainability frameworks into model development processes.

In addition to summarizing current applications, this review also discusses major methodological limitations affecting the translation of AI models into cardiovascular clinical practice.

Furthermore, a crucial aspect worth considering when discussing the limitations of current research on innovative technologies in cardiology is the limited data on the actual impact on clinical outcomes and standardization of methodology. The literature on three-dimensional (3D) printing in cardiology frequently highlights the potential of this technology—from accurate reconstruction of cardiac anatomy, through procedure planning and surgical simulations, to patient and staff education—however, most of the available evidence comes from case reports and small clinical series, limiting the ability to assess its actual impact on treatment outcomes and therapeutic decisions. Studies emphasize that 3D printing can provide accurate anatomical models, support planning of TAVR procedures or complex procedures for congenital heart defects, and serve as a teaching tool. However, large, multicenter prospective studies assessing the clinical effects and cost-effectiveness of this technology in everyday clinical practice are still lacking. In the context of 3D printing, it’s also worth highlighting technological limitations, such as static image representation (lack of dynamic systolic and diastolic heart function) and the lack of standards for materials and processes, which hinders comparison of results between centers [158].

## 4. Discussion

### 4.1. Radiomics and Deep Learning in Quantitative Cardiac Imaging

The development of machine learning techniques has radically transformed the processing and analysis of large, multidimensional datasets, giving rise to the field of radiomics in medical imaging. Radiomics enables the quantitative extraction of hundreds, even thousands, of features from medical images, converting visual information into data suitable for exploration using artificial intelligence tools. This allows not only the detection of subtle structural and functional changes, invisible to the naked eye, but also the creation of predictive models for disease progression and clinical risk [159]. Among the extracted features are morphological, intensity, textural, and fractal aspects [160]. Texture analysis (TA), a subset of radiomics that focuses on quantifying tissue patterns and heterogeneity, plays a particularly important role. TA uses machine learning algorithms to examine the spatial relationships between neighboring pixels, allowing for the determination of imaging parameters that go beyond traditional visual interpretation [161].

In cardiology, radiomics is gaining importance in the diagnosis and prognosis of cardiovascular diseases such as coronary artery disease, heart failure, and structural abnormalities of the myocardium. Integrating radiomics with deep learning enables automated segmentation of cardiac structures, identification of tissue abnormalities, and creation of predictive models of clinical risk. This enables a more precise, quantitative approach to assessing microscopic and functional changes in the heart, supporting therapeutic decisions and predicting the course of the disease at the individual patient level. Radiomics combined with AI is therefore becoming a powerful tool in precision cardiology, offering the opportunity for earlier detection and improved patient monitoring [162,163].

### 4.2. Deep Learning in CMR: LGE Quantification, 4D Flow Analysis and Image Reconstruction

In recent years, several researchers have focused on the use of DL to precisely measure myocardial late enhancement (LGE). Fahmy et al. [164] were the first to use two-dimensional CNNs to quantify enhancement in patients with hypertrophic cardiomyopathy (HCM). The development of three-dimensional LGE imaging (3D MRI) has enabled isotropic, high-resolution reconstruction of scar geometry, which in turn allowed Zabihollahy et al. [163] to develop a 3D CNN for automatic segmentation of left ventricular scar.

Subsequently, Fahmy et al. [164] presented a 3D CNN trained on a large, manually segmented, multi-center dataset that enabled accurate, automatic quantification of LGE scar at a level comparable to expert analysis. Together, these studies demonstrate that both 2D and 3D DL models offer high accuracy in LGE analysis, although their evaluation in broad clinical practice still requires further investigation.

Besides scar analysis, neural networks show promising applications in 4D flow phase correction in MRI. Berhane et al. [165] developed a U-Net network to detect and correct aliased voxels in 4D flow images, achieving Sørensen–Dice values in the range of 0.89–0.99, outperforming conventional aliasing detection algorithms. You et al. [166] demonstrated that CNNs can automatically correct phase errors in 4D MRI data using a multi-channel 3D U-Net, providing results comparable to manual corrections used in traditional software. Such algorithms reduce the need for manual intervention in phase error correction, which currently requires extensive technical expertise rarely available to clinicians interpreting cardiac images.

Recent advances in DL have significantly improved the analysis of cardiovascular images across various modalities. In echocardiography, lightweight convolutional and transformer networks, such as EchoNet-Dynamic and UltraViT, enable fast and accurate ventricular segmentation and cardiac function assessment [134]. In PET/CT, U-Nets with attention mechanisms improve perfusion quantification, lesion localization, and noise reduction while reducing radiation dose [107]. In MRI/CMR, nnU-Nets and U-Nets with transformers achieve near-expert performance in myocardial segmentation and abnormality detection [108], and in NMR spectroscopy, DL enables precise metabolite quantification and biomarker identification [109].

Deep learning methods have increasingly been applied to CMR imaging to support automated diagnosis of cardiovascular diseases. Early studies demonstrated that neural networks can detect subtle imaging features that may not be easily recognized during routine visual assessment. In particular, convolutional neural networks trained on cine MRI images were able to identify patterns associated with chronic myocardial infarction even without the use of contrast agents, suggesting that AI can extract clinically relevant information from standard imaging sequences and potentially reduce the need for additional contrast-enhanced examinations [167].

Subsequent research expanded the application of deep learning to the diagnosis of specific cardiomyopathies. For example, AI-based models have been successfully used to identify imaging signatures of cardiac amyloidosis from CMR data. By analyzing structural and functional myocardial characteristics, deep learning algorithms were able to differentiate amyloidosis from other cardiomyopathies with high diagnostic accuracy. These findings indicate that AI can support clinicians in recognizing rare or complex cardiac diseases and may facilitate earlier and more precise diagnosis [168].

More recently, large-scale studies have demonstrated the potential of artificial intelligence to support comprehensive cardiovascular disease screening using cardiac magnetic resonance imaging. AI-enabled CMR analysis can integrate multiple imaging features and automatically detect patterns associated with a broad spectrum of cardiovascular conditions. Such approaches may improve the efficiency of screening programs, enhance risk stratification, and enable earlier identification of patients requiring further clinical evaluation or treatment [169].

Despite these promising developments, important challenges remain regarding the generalizability and robustness of medical AI models. One major concern is the phenomenon of “shortcut learning,” where algorithms rely on unintended correlations in the training data rather than clinically meaningful features. This may lead to reduced performance when models are applied to new patient populations or imaging environments. Therefore, careful validation strategies and methods for assessing model generalization are essential to ensure safe and reliable implementation of AI systems in clinical practice [170].

Advances in deep learning techniques for MRI image reconstruction are leading to real clinical benefits in accelerating examinations without sacrificing diagnostic quality. A systematic evaluation of DL-based reconstruction in cardiac image acquisition demonstrated that accelerated cine bSSFP sequences obtained from undersampled k-space using DL maintain similar volumetric indices (e.g., ventricular volumes) as fully sampled sequences, even at high acceleration factors, confirming their potential to shorten examination time without significantly compromising the diagnostic quality of volumetric data [171].

In prospective studies, DL-based reconstruction techniques significantly shorten overall MRI acquisition time while maintaining comparable image quality and accuracy of cardiac function measurements. In one such study, cine sequences with DL reconstruction achieved over 50% reduction in scanning time compared to reference sequences, without statistically significant differences in image quality and volumetric results of the left ventricle [172]. Subsequent studies confirmed that DL-accelerated cine sequences in CMR reduce examination time even while maintaining comparable assessment of functional parameters of the right and left ventricle and maintaining acceptable image quality, which is crucial for transferring these methods to everyday clinical practice [173].

Moreover, recent analyses indicate that compressed AI-assisted sensors (CSAI) can improve not only cine sequences but also other important cardiac MRI protocols, such as T2 STIR and LGE, while reducing acquisition times by more than half, increasing SNR and CNR compared to conventional SENSE reconstruction. These results confirm that integrating DL into CMR image reconstruction can improve the quality and efficiency of clinical trials while maintaining the consistency of quantitative diagnostic parameters [48].

### 4.3. AI-Based Predictive Models and Multimodal Data Integration in Cardiovascular Medicine

The integration of multimodal data is one of the most promising strategies for developing artificial intelligence systems that support clinical decision-making in cardiovascular diseases. Combining information from imaging (CCTA, CMR, echocardiography, PET), clinical data, laboratory parameters, ECG signals, and, increasingly, omics data allows for the creation of more comprehensive representations of a patient’s phenotype. Unlike single-modality models, data fusion strategies enable the simultaneous consideration of anatomical, functional, and molecular features, leading to more accurate risk stratification and more personalized therapeutic decisions. In the context of coronary artery disease, integrating atherosclerotic plaque features obtained from CCTA with hemodynamic parameters (FFR-CT), inflammatory markers, and clinical data increases the accuracy of predicting cardiovascular events. Currently, the integration of multimodal data using AI is one of the most effective methods for supporting the diagnosis and management of cardiovascular diseases.

Artificial intelligence is also playing an increasingly important role in cardiac electrophysiology, revolutionizing both the diagnosis and treatment of arrhythmias. Thanks to its ability to analyze large and complex datasets, AI enables the detection of subtle patterns that are not visible in traditional clinical assessments. In clinical practice, AI-based tools support the interpretation of ECG signals, the identification of supraventricular arrhythmias such as atrial fibrillation and flutter, and complex ventricular arrhythmias, including ventricular tachycardia and long QT syndrome. AI is also used in interventional procedures, improving electroanatomical mapping, shortening procedure times, and supporting personalized post-ablation care planning. Despite clear benefits in terms of diagnostic accuracy and procedural efficiency, the implementation of AI in electrophysiology requires further development of data safety standards, ethical transparency, and standardization of implementation procedures to fully leverage its potential in daily cardiology practice [174].

A review by Tran et al. shows that AI-assisted multimodal imaging, combining CCTA, MRI, and echocardiography, can improve diagnostic accuracy and risk stratification, although the implementation of these technologies in routine clinical practice faces technical, infrastructural, and validation barriers [175]. Multimodal AI approaches enable the simultaneous use of various data sources (e.g., imaging, EHR, biochemical parameters) to better predict disease progression and determine prognosis. The integration strategy employs early, intermediate, and late data fusion approaches [176,177]. Milosevic et al. emphasize that while integration yields promising results, its implementation in real-world clinical settings remains limited [178].

Ghenciu et al. highlighted the growing potential of AI-based retinal image analysis in predicting cardiovascular risk factors and events [179]. This study employed a hybrid predictive strategy based on a one-dimensional convolutional neural network (1D-CNN), integrating data from online surveys with datasets such as UCI, MIMIC, and MIT-BIH, achieving promising classification results [180]. Comparative analyses of classical ML models (NB, SVM, RF, LR, LDA) demonstrated high accuracy of Random Forest in structured clinical data. Hybrid DNN + CBR approaches achieved very high accuracy in predicting CAD (96.2%) [181,182,183,184,185]. Furthermore, the use of ML in predicting hepatocellular carcinoma confirms the scalability and versatility of ML methods in clinical analysis [186].

Deep learning models used in CCTA demonstrate high specificity and predictive value in detecting significant coronary artery stenosis [187,188]. AI also improves prognostic stratification across various imaging modalities [189], including applications in low- and middle-income countries [190].

The development of digital cardiology has accelerated in recent years thanks to advances in high-resolution imaging, wearable device technology, and computer analysis. The integration of large-scale, multi-source imaging and clinical data enables the creation of more precise prognostic and diagnostic algorithms, supporting a personalized medicine approach. Such datasets increase the robustness of models, allow for the detection of subtle disease patterns, and enable more accurate risk stratification for individual patients. At the same time, the development of AI tools in various areas of cardiology, including the analysis of ophthalmic data and the integration of omics data, confirms the growing potential of this technology in the broad diagnosis and prediction of cardiovascular events.

To facilitate comparison and highlight potential applications of specific AI architectures in cardiology, Table 4 provides an overview of the most commonly used models, along with their data types, advantages, limitations, and interpretability.

A comparative analysis of available AI architectures indicates that there is no single universal model that is optimal for all cardiological applications. Predictive performance depends primarily on the type of input data, the complexity of the clinical problem, and the expected level of model interpretability. In medical imaging analysis, deep learning models, particularly CNNs, achieve the highest performance due to their ability to automatically extract spatial features. For sequential data, such as ECG signals or long-term telemetry data, RNN and LSTM architectures demonstrate an advantage, enabling the modeling of temporal dependencies. In contrast, for the analysis of structural and laboratory data, classical machine learning models, such as Random Forest and Gradient Boosting, remain particularly useful due to their high predictive stability and greater interpretability. Multimodal integration strategies, which combine imaging, clinical, and biochemical data, are also becoming increasingly important, allowing for a more precise assessment of cardiovascular risk and the personalization of therapeutic decisions. At the same time, the further development of clinical AI applications requires improved external validation of models, data standardization, and broader implementation of explainable artificial intelligence methods, which enhance the safety and trustworthiness of clinical decision support systems [207,208,209].

### 4.4. Telemonitoring and Wearable Technologies

Telemonitoring and digital technologies in the form of wearable devices represent a significant expansion of AI-supported cardiac care, going beyond conventional imaging and hospital-based diagnostics. Remote blood pressure monitoring, smartphone apps, and networked wearable devices increasingly enable long-term assessment of cardiovascular parameters in real-world settings, improving both early disease detection and long-term adherence to treatment recommendations. Beyond conventional telemonitoring, wearable devices equipped with sensors for continuous electrocardiographic monitoring, blood pressure estimation, photoplethysmographic analysis, and physical activity tracking generate large volumes of longitudinal physiological data that are particularly well suited to machine learning analysis. For example, the Fitbit Heart Study demonstrated the feasibility of photoplethysmography-based atrial fibrillation detection in over 455,000 participants using a wrist-worn wearable device, achieving a positive predictive value of 98.2% for concurrent atrial fibrillation when confirmed by ECG patch monitoring [210]. These findings support the growing role of consumer wearable technologies as scalable tools for early arrhythmia detection in large populations.

The latest data from the randomized ESH CARE trial showed that monitoring patients with uncontrolled hypertension using an app significantly improved blood pressure control compared to standard care: after 6 months, ambulatory blood pressure targets were achieved in 78.2% of patients in the intervention group compared to 50% in the standard group, with greater reductions in both office and ambulatory blood pressure. These results suggest that digital telemonitoring can enhance patient engagement, facilitate earlier treatment adjustments, and complement AI-based predictive models by providing continuous physiological data outside of traditional clinical settings [211]. These findings further underscore the clinical utility of mobile health interventions in hypertension management, indicating that app-assisted monitoring can improve adherence to antihypertensive therapy, facilitate virtual follow-up, and support more precise achievement of blood pressure targets through continuous remote supervision [212]. Integrating AI-driven analytics with such telemonitoring platforms represents a promising direction for precision management of cardiovascular risk factors.

Meanwhile, the Internet of Things in the treatment of cardiovascular diseases opens up new possibilities for remote patient monitoring, early detection of arrhythmias, continuous analysis of signals such as ECG or heart rhythm, and support for home care, potentially transforming the model of cardiac care from hospital-centric to a more distributed and personalized approach [213,214]. Combining IoT with advanced AI models enables real-time analysis of large datasets, but also introduces challenges related to system interoperability, data security, and integration with existing clinical environments [215]. More broadly, recent scientific statements indicate that remote monitoring platforms integrating symptoms, vital signs, and cardiac filling pressure measurements with AI-driven analytics may enable personalized real-time risk assessment and timely intervention, particularly in patients with heart failure [216]. Such systems may improve early identification of hemodynamic deterioration, medication non-adherence, and subclinical arrhythmias before hospital admission becomes necessary.

In recent years, there has been growing interest in the application of generative artificial intelligence (AI) models to the analysis of time-varying biomedical data, which represents a natural extension of traditional methods for predicting and monitoring health status. The trajectories of health parameters, such as heart rate, blood pressure, and biomarker dynamics, pose a challenge due to their complex, multimodal, and long-term relationships. Traditional statistical models and classical machine learning methods often fail to fully capture these relationships, whereas generative AI approaches can represent complex data distributions and patterns with minimal prior assumptions. In a review by He, Rosemary, et al., 155 studies on the application of generative AI models to time-series health data were reviewed, covering both single and multimodal data sources, including structured and unstructured clinical data, physiological signals, medical imaging, and multi-omics data. The authors presented the basic concepts of these models, currently used algorithms, and their potential applications in clinical practice, while highlighting limitations and directions for further research. Generative AI models offer promising tools for predicting the dynamics of health parameters, personalizing treatments, and simulating clinical scenarios, facilitating the integration of data from imaging, telemonitoring, and laboratory tests, which further enhances the ability to make decisions based on personalized medicine [217].

### 4.5. Translational Barriers, Ethical Considerations, and Future Perspectives

Despite promising evidence, key challenges in translating AI into routine clinical practice remain. Barriers include limited diversity of training data, heterogeneity in imaging protocols, system interoperability issues, and limited interpretability. Therefore, future research should focus on prospective, multi-center validation of algorithms, development of reporting standards, and integration into clinical workflows.

In parallel with technological advances, ethical aspects of AI implementation are increasingly emphasized. DL models often function as a “black box,” limiting interpretability and potentially affecting informed consent and clinical accountability. Literature highlights the need for XAI tools and interpretive frameworks to improve transparency and trust.

Algorithmic fairness and patient data protection represent additional critical issues. Underrepresentation of certain populations may lead to biased performance and unequal healthcare outcomes. Furthermore, integration of large clinical and imaging datasets raises privacy challenges under legal frameworks such as GDPR. Techniques such as federated learning and privacy-preserving encryption algorithms are necessary to enable secure distributed learning without violating patient confidentiality [218,219,220].

## 5. Conclusions

The evidence presented in this review indicates that artificial intelligence and deep learning have moved beyond proof-of-concept applications and are now demonstrating clinically relevant performance across multiple domains of cardiovascular imaging. In cardiac CT, CMR, echocardiography, and PET, AI-based algorithms achieve diagnostic accuracy comparable to expert readers, while significantly reducing analysis time and operator dependency. In particular, deep learning-based segmentation, plaque characterization, myocardial scar quantification, and functional assessment have shown reproducibility and robustness across diverse datasets.

Deep learning-accelerated image reconstruction in CMR enables substantial shortening of acquisition time without compromising volumetric measurements or diagnostic image quality, supporting its integration into routine clinical workflows. Similarly, multimodal AI approaches that combine imaging, clinical, and laboratory data consistently improve risk stratification compared to single-modality models, highlighting the added value of data fusion strategies.

However, despite strong diagnostic performance, widespread clinical implementation requires prospective, multicenter validation and standardized reporting frameworks. Model interpretability, external generalizability, and mitigation of algorithmic bias remain essential prerequisites for safe deployment. Ethical safeguards—including data protection strategies and transparent model governance—are critical to maintaining clinical accountability.

## Figures and Tables

**Table 1:** Performance comparison of ML/DL models versus expert or traditional methods across cardiovascular and cerebrovascular diagnostics. Studies include ischemic stroke segmentation and infarct prediction (CTP, MRI DWI/ADC) and coronary artery disease risk stratification (CCTA with clinical data). AI/ML, artificial intelligence/machine learning; CTP, CT perfusion; DWI, diffusion-weighted imaging; ADC, apparent diffusion coefficient; CCTA, coronary CT angiography; AUC, area under the curve; ICC, intraclass correlation coefficient.

No.	Disease	Modality	AI/ML Model	Expert/Traditional Method	AI/ML Performance Indicators	Expert Performance Indicators	Conclusions	Article
1	Ischemic stroke	CTP + Tmax	MultiRes U-Net	Radiologist	Dice = 0.68	Dice ≈ 0.50	AI outperforms experts in stroke core segmentation, with better reproducibility of results	[14]
2	Ischemic stroke	MRI DWI + ADC	U-Net	MRI (manual segmentation)	Dice up to 0.97 (DWI + ADC); interobserver ICC > 0.98	Dice ≈ 0.74 (DWI alone)	Integration of DWI + optimized ADC thresholds in DL improves segmentation accuracy and interobserver consistency	[15]
3	Ischemic stroke	MRI DWI	CNN (attention-gated)	ADC thresholding	Median AUC = 0.91 (IQR: 0.84–0.96)	Lower volumetric accuracy vs. CNN	AI enables earlier prediction of final infarct volume without additional PWI	[18]
4	Coronary artery disease	CCTA	ML (XGBoost)	Cardiologist/visual assessment	AUC = 0.92 (95% CI 0.89–0.94)	AUC = 0.84	AI better predicts ischemia and hemodynamically significant stenosis than visual assessment	[20]
5	Coronary artery disease	CCTA + clinical data	ML (LogitBoost)	Framingham Risk Score	AUC = 0.79	AUC = 0.61	ML integrated with clinical data outperforms classic risk scores in predicting all-cause mortality	[23]
6	CAD	CCTA + clinical data	Random survival forests (time-to-event ML)	Cox proportional hazards model	C-index = 0.74	C-index = 0.71	ML models provide better prognostic performance than traditional Cox regression models	[21]

**Table 2:** Representative AI techniques applied across stages of cardiovascular image processing, organized by imaging modality (CMR, CCTA), algorithmic approach (CNN, U-Net, GAN, DL iterative reconstruction, nnU-Net), processing task (reconstruction acceleration, dose reduction, contrast enhancement, segmentation), and clinical application (risk prediction).

Stage	Modality	AI Technique	Clinical Application	Clinical Benefit	Study
Reconstruction	CMR	CNN, U-Net, GAN	Accelerating acquisition	↓ examination time, ↑ SNR	[48]
Reconstruction	CCTA	DL iterative recon	Dose reduction	↓ radiation	[78]
Improvement	CMR	DL denoising	LGE contrast enhancement	↑ fibrosis detection	[79]
Analysis	CMR	nnU-Net	LV/RV segmentation	Measurement automation	[80]
Analysis + XAI	CMR/CT	CNN + SHAP	MACE prediction	Clinical confidence	[61]

**Table 3:** Comparison of multimodal data fusion strategies in cardiovascular AI. Three approaches, early fusion (feature-level concatenation), late fusion (decision-level aggregation), and hybrid fusion (combined feature- and decision-level integration), are compared across definition, architecture, data combination method, interpretability, advantages, limitations, and clinical examples. Conceptual framework based on Yang et al. [112], with examples added by the authors.

Feature/Criterion	Early Fusion (Data Level)	Late Fusion (Decision Level)	Hybrid/Intermediate Fusion (Function Level)
Definition	Data or features from different modalities combined at the input of a single model prior to extraction and classification.	Each modality is analyzed separately, and their decisions/predictions are combined at the end.	Features from different stages of extraction are combined in intermediate representations (latent space).
Level of integration	Low → directly on input data	High → at the model decision level	Average → at the level of internal characteristics
Architecture model	One model with multi-channel input	Separate models → aggregation of results	Multistream networks + attention mechanisms
Interactions between modalities	Can capture low-level relationships	Limited to decisions	Good → allows for deeper relationships
Resistant to missing data	Low resistance	High resistance	Average—requires design
Computational complexity	High	Moderate	High
Interpretability (without XAI)	Limited	Better	Moderate
Examples of research/applications	Multimodal ECG + EMR joints for PCI prognosis—the early/joint fusion model achieved better results than unimodal models [80]	Late fusion combining images and clinical data in the classification of pulmonary embolism with high AUROC [110]	Hybrid imaging integrating images, features, and clinical data to improve cardiac disease detection and other applications—although specific cardiology work is still evolving [111]
Advantages	Enables learning of low-level relationships	Modular analysis of each modality, flexible	Preserves dependencies and redundancies in features
Disadvantages	Requires data standardization, difficult modality synchronization	Does not teach feature-level interactions	Complex architecture, higher data requirements

**Table 4:** A Comparison of Artificial Intelligence Architectures Used in Cardiovascular Data Analysis: Characteristics, Advantages, Limitations, Interpretability, and Potential Clinical Applications.

AI Architecture	Primary Modality	Typical Application	Performance Highlights	Interpretability	Limitations/Trade-Offs	References
CNN (2D/3D)	CMR, CCTA, Echo	Image classification, lesion detection, disease diagnosis	AUC = 0.96 for cardiac amyloidosis classification from CMR (sensitivity 94%, specificity 90%); accuracy 96.8% for echo view classification with external validation	Low (black-box); partially addressed by Grad-CAM	Requires large labeled datasets; limited generalizability across scanners/protocols	[191,192]
U-Net/nnU-Net	CMR, PET/CT	Cardiac chamber segmentation, phase correction, perfusion quantification	Average Dice ~0.916 on ACDC benchmark (LV ~0.97, myocardium ~0.91, RV ~0.94 at end-diastole); Dice 0.94/0.89/0.90 (LV/myo/RV) in free-breathing CMR	Low–Moderate; architecture is transparent but learned features are not	Sensitive to domain shift; most validated on single-center data	[110,193]
Transformer-based (e.g., Swin-Unet)	CMR, Echo	Cardiac segmentation, functional assessment	Average Dice 90.00% on ACDC, outperforming U-Net (87.55%) and TransUNet (89.71%); Dice 92.92% for echo LV segmentation	Low; attention maps offer partial insight	Higher computational cost; fewer cardiovascular-specific validation studies; do not universally outperform CNNs	[194,195]
CNN-LSTM hybrid	Echo, CMR (cine)	Temporal sequence analysis, EF estimation from video	MAE 5.74% for EF estimation from echo video (outperforming RNN, SVR, linear regression); direct volume prediction from cine MRI without explicit segmentation	Low; temporal reasoning difficult to explain	Small validation cohorts; limited to specific acquisition protocols	[196,197]
GAN	CMR	Image reconstruction, super-resolution, synthetic data augmentation	PSNR improvement +9.57 dB over conventional SENSE reconstruction; superior PSNR/SSIM for cardiac MRI super-resolution across multiple upscaling factors	Very low; generative process is opaque	Risk of hallucinated features; difficult to validate clinically; limited prospective studies	[198,199,200]
Random Forest/Gradient Boosting	Clinical + lab data, ECG	Risk prediction, event forecasting, mortality prediction	GB AUC = 0.761 vs. ACC/AHA AUC = 0.728 for 10-year CVD risk (n = 378,256); XGBoost AUC = 0.916 for HF mortality prediction	High; feature importance readily available (SHAP)	Cannot process raw imaging data; limited to structured inputs	[201,202]
Multimodal fusion (early/late/hybrid)	Echo + CMR + PET/CT + clinical	Comprehensive risk stratification, PE detection, perioperative MACE prediction	Late fusion AUROC = 0.947 for PE detection; multimodal CCTA AUROC = 0.82 vs. 0.69 single-modality for perioperative MACE	Varies by fusion strategy; late fusion more modular	Data heterogeneity; missing modality handling; limited prospective validation	[113,203,204]
XAI-enhanced DL (Grad-CAM, SHAP, LIME)	SPECT, Echo, ECG	Interpretable diagnosis, clinician decision support	AUC = 0.83 vs. expert reader 0.71 for CAD detection from SPECT with Grad-CAM maps (n = 3578, multicenter); accuracy 98.9% for MI detection from ECG with Grad-CAM	High (by design); identifies key predictive features validated against clinical knowledge	XAI explanations not yet standardized; may oversimplify model reasoning	[205,206]

## Data Availability

No new data were created or analyzed in this study.
